# Identifying determinants of spatial agglomeration of healthcare resources by using spatial econometric methods: a longitudinal study in China

**DOI:** 10.3389/fpubh.2024.1351849

**Published:** 2024-05-28

**Authors:** Enhong Dong, Xiaoting Sun, Yueming Xi, Yijia Wang, Tao Wang, Weimin Gao

**Affiliations:** ^1^School of Nursing and Health Management, Shanghai University of Medicine and Health Science, Shanghai, China; ^2^Institute of Health Yangtze River Delta, Shanghai Jiao Tong University, Shanghai, China; ^3^Shanghai Tenth Peoples Hospital Affiliated to Tongji University, Shanghai, China; ^4^School of Public Health, University of Hangzhou Normal University, Hanzhou, China; ^5^School of Basic Medicine, Capital Medical University, Beijing, China; ^6^Shanghai Tongji Hospital, Tongji University School of Medicine, Shanghai, China; ^7^School of Nursing, Kunming Medical University, Kunming, China

**Keywords:** determinants, healthcare resources, agglomeration, spatial econometric methods, China

## Abstract

**Background:**

Healthcare resources are necessary for individuals to maintain their health. The Chinese government has implemented policies to optimize the allocation of healthcare resources and achieve the goal of equality in healthcare for the Chinese people since the implementation of the new medical reform in 2009. Given that no study has investigated regional differences from the perspective of healthcare resource agglomeration, this study aimed to investigate China’s healthcare agglomeration from 2009 to 2017 in China and identify its determinants to provide theoretical evidence for the government to develop and implement scientific and rational healthcare policies.

**Methods:**

The study was conducted using 2009–2017 data to analyze health-resource agglomeration on institutions, beds, and workforce in China. An agglomeration index was applied to evaluate the degree of regional differences in healthcare resource allocation, and spatial econometric models were constructed to identify determinants of the spatial agglomeration of healthcare resources.

**Results:**

From 2009 to 2017, all the agglomeration indexes of healthcare exhibited a downward trend except for the number of institutions in China. Population density (PD), government health expenditures (GHE), urban resident’s disposable income (URDI), geographical location (GL), and urbanization level (UL) all had positive significant effects on the agglomeration of beds, whereas both *per capita* health expenditures (PCHE), number of college students (NCS), and maternal mortality rate (MMR) had significant negative effects on the agglomeration of institutions, beds, and the workforce. In addition, population density (PD) and *per capita* gross domestic product (PCGDP) in one province had negative spatial spillover effects on the agglomeration of beds and the workforce in neighboring provinces. However, MMR had a positive spatial spillover effect on the agglomeration of beds and the workforce in those regions.

**Conclusion:**

The agglomeration of healthcare resources was observed to remain at an ideal level in China from 2009 to 2017. According to the significant determinants, some corresponding targeted measures for the Chinese government and other developing countries should be fully developed to balance regional disparities in the agglomeration of healthcare resources across administrative regions.

## 1 Introduction

Healthcare resources are necessary for individuals to maintain their health. Thus, these resources should be rationally allocated to ensure sustainable health services. Moreover, to ensure the performance of the health system, people’s growing and diversified needs for health services should be effectively met. China’s vastness and diversity, imbalanced development and variety of economic activities, population growth, cultural differences, geography, and transportation conditions across regions, as well as the fragmentation of the urban–rural dual system structure [0–0], have resulted in the maldistribution of healthcare resources. Differences in healthcare resource allocation between and within regions can be observed in China.

The Chinese government has implemented policies to optimize the allocation of healthcare resources and achieve the goal of equality in healthcare for the Chinese people. Since the implementation of the new medical reform in 2009, the government has increased investment in healthcare resources, particularly prioritizing financial support in the central and western regions, expanding medical insurance coverage, improving primary health services, developing a hierarchical diagnosis and treatment system, and reducing health expenditures *per capita* and differences in healthcare resource allocation between developed and underdeveloped regions.

By employing the data envelopment analysis approach, Sha et al. (1) observed regional differences in the efficiency of healthcare resource allocation in China, with the allocation being more inefficient in five cities in the Shanxi province of China. Socioeconomic factors, including income, education, and insurance, were identified as the main determinants of regional differences in healthcare resource allocation. Gu J (2) identified population, *per capita* gross domestic product (GDP), number of urban employees, level of commercial and trade development, and proportion of the agricultural area as contributors to the allocation of healthcare resources in some counties in China. However, Tuvia et al. demonstrated that inequality in socioeconomic factors resulted in inequality in income, employment, and health investment, thus affecting the fairness of the distribution of health resources (3). Furthermore, Qiong reported that income, medical insurance, and health service supply and consumption demand were the determinants of differences in healthcare resource allocation between urban and rural areas (4). Moreover, Xie et al. demonstrated that urban residents’ medical insurance and inequality in household income were the determinants of differences in healthcare resource allocation between urban and rural areas (5). Li et al. posited pro-wealthy inequality in the utilization of maternal health services in the rural areas of western China, and this inequality was identified as being associated with factors such as income, education level, and geographical transportation conditions (6). Although many studies have examined the time trend of differentiation in healthcare resource allocation in various regions of China by using many methods and healthcare resource indicators (e.g., health expenditures and numbers of health institutions beds and technicians), no study has investigated regional differences from the perspective of healthcare resource agglomeration and the spatial spillover effect of healthcare agglomeration by considering the spatial heterogeneity of these resources. In fact, agglomeration–the process by which economic activities cluster together in geographic space–is a complex phenomenon influenced by a variety of factors. It often occurs in healthcare resources within specific geographic areas or institutions. This phenomenon is influenced by a complex interplay of determinants, which can be analyzed through different theoretical lenses, such as Anderson’s behavioral model, which was originally designed to explain and predict healthcare utilization. The model can also provide insights into the factors that contribute to the agglomeration of healthcare resources. According to this theoretical model, three types of factors contribute to healthcare utilization, namely predisposing characteristics (e.g., population density and age distribution), enabling resources (e.g., insurance coverage, healthcare facilities, and human resources), and need factors (e.g., high demand for certain types of care, self-reported health status, and population mortality); these factors would all lead to the concentration of healthcare resources (7). Scholars have posited the agglomeration of healthcare resources is a multifaceted phenomenon influenced by economic principles, demographic factors, technological advancements, and policy decisions (8). Therefore, understanding these determinants is crucial for policymakers and healthcare administrators as they work to optimize the distribution of healthcare resources to best meet the needs of the population.

China has gradually overcome geographical and administrative constraints by increasing investment in health services and expanding the number of healthcare resources across various administrative divisions. This type of agglomeration can demonstrate the spatial autocorrelation among various regions in China. Therefore, given the implementation of the new medical reform and the rapid development of health services in China, regional differences in the agglomeration of healthcare resources and the spatial spillover effect of healthcare agglomeration should be investigated.

Using spatial econometric methods and longitudinal panel data (2010–2017), this study explored China’s healthcare agglomeration and identified its determinants to provide theoretical evidence for the government to develop and implement scientific and rational healthcare policies.

## 2 Methods

### 2.1 Data sources

Secondary data for this study were obtained from the China Health Statistical Yearbook and China Statistical Yearbook for 31 provinces (municipalities or autonomous regions) in China from 2010 to 2018 (Considering the one-year time delay nearly existed in publishing Chinese official annual yearbooks). These yearbooks are published officially by the National Health Commission and the National Bureau of Statistics, and the raw data supporting the conclusions of this study will be made available from the corresponding author upon reasonable request.

In this study, the numbers of health institutions, beds, doctors, technicians, and nurses were included as healthcare resource indicators. Data on maternal mortality rate (MMR), rate of born-baby weight less than 2. 5 kg (RBWL25), perinatal mortality rate (PMR), government health expenditures (GHE), out-of-pocket (OOP) expense, and *per capita* health expenditures (PCHE) were retrieved from the China Health Statistical Yearbook (2009–2017), and those on the population density (PD), *per capita* gross domestic product (PCGDP), number of insured persons (NI), number of college students (NCS), urban residents disposable income (URDI), farmers net income (FNI), and urbanization level (UL) were retrieved from the China Statistical Yearbook (2010–2018); these factors were included as contributing variables. The description of the variables of interest and their units of measurement in the study can be seen in Table 1.

**Table 1:** Description of the variables of interest and its units of measurement in the study.

Variables	Description	Unit	Type
IA	Instituions agglomeration	None	Categorical variable
BA	Beds agglomeration	None	Categorical variable
DA	Doctor agglomeration	None	Categorical variable
TA	Technicians agglomeration	None	Categorical variable
NA	Nurses agglomeration	None	Categorical variable
PD	Population per unit land area	None	Categorical variable
MMR	The annual number of female deaths per 100,000 live births	Persons per 100,000 live births	Categorical variable
RBWL25	The annual number of live-born infants weighing less than 2,500 g per 100 live births	Percent	Categorical variable
PMR	The annual number of stillbirths and deaths in the first week of life per 1,000 total births	Per 1,000 births	Categorical variable
GHE	Annual government health expenditures	100,000,000Yuan	Categorical variable
OOPE	Out-of-pocket expenditure on health	Yuan	Categorical variable
PCHE	*Per capita* health expenditures	Yuan	Categorical variable
PCGDP	*Per capita* gross domestic product	Yuan	Categorical variable
NI	The number of Insured population	10,000 persons	Categorical variable
NCS	The number of college students	Persons	Categorical variable
URDI	Annual Urban Residents’ Disposable Income	Yuan	Categorical variable
FNI	Annual Farmers ‘Net Income	Yuan	Categorical variable
GL	Geographical Location		Continuous variable
UL	Urbanization Level	per cent	Categorical variable

### 2.2 Statistical methods

Based on the literature on location entropy, we used the agglomeration index to evaluate the degree of regional differences in healthcare resource allocation, which is represented by CYHR. CYHR refers to the total health resources in each province divided by the total population in each province. CYHR_j_ refers to the total number of health resources available to the total population for the healthcare resource indicator j. CYHJJ_ij_ is an index of regional differences in agglomeration and is calculated as follows:


(1) $$CYHJJ_{\mathrm{ij}}=\left(\frac{CYH_{ij}/P_{ij}}{CYH_{j}/P}\right),i=1,2,\ldots ,31;j=1,2,...5$$

In Formula (1), where CYHJJ_ij_ represents the agglomeration index of regional differences in unit i for the jth healthcare resource indicator, CYH_ij_ represents the resource allocation in a province i for the jth index, P_ij_ refers to the total population in unit i for the jth indicator, CYH_j_ refers to the jth indicator in one area, P represents the total population in one area, and j refers to one healthcare resource indicator among the five total indicators. According to the definition of the agglomeration index, if CYHJJ_ij_ is>1, resource agglomeration in region i for the jth indicator is higher than the average national level, indicating a higher allocation of resources in region i for the jth indicator than in regions with the average national level. Otherwise, a CYHJJ_ij_ value of <1 indicates a lower allocation of resources in region i for the jth indicator than in regions with the average national level.

First, we applied a multivariable linear regression model to investigate the factors associated with a quantitative variable as follows:


(2) $$\begin{array}{l} {\mathrm{CYHJJ}}_{\mathrm{ij}}=\beta_{0}+\beta_{1}PD_{\_ij}+\beta_{2}MMR_{\_ij}+\beta_{3}RBWL25_{\_ij}\\ \;\;\;\;\;\;\;\;\;\;\;\;\;\;\;\;\;\;\;\;\;\;\;\;\;+\beta_{4}PMR_{\_ij}+\beta_{5}GHE_{\_ij}+\beta_{6}OOPE_{\_ij}\\ \;\;\;\;\;\;\;\;\;\;\;\;\;\;\;\;\;\;\;\;\;\;\;\;\;+\beta_{7}PCHE_{\_ij}+\beta_{8}NI_{\_ij}+\beta_{9}NCS_{\_ij}\\ \;\;\;\;\;\;\;\;\;\;\;\;\;\;\;\;\;\;\;\;\;\;\;\;\;+\beta_{10}PCGDP_{\_ij}+\beta_{11}URDI_{\_ij}+\beta_{12}FNI_{\_ij}\\ \;\;\;\;\;\;\;\;\;\;\;\;\;\;\;\;\;\;\;\;\;\;\;\;\;+\beta_{13}GL_{\_ij}+\beta_{14}UL_{\_ij}+\varepsilon_{\_ij} \end{array}$$

In Formula (2), where *CYHJJ_ij_* refers to the agglomeration index of regional differences in healthcare resources, *i* refers to the number of provinces in China, and *j* refers to the type of healthcare resources: healthcare institutions, beds, doctors, technicians, and nurses. β_i_ refers to the regression coefficient of the explanatory variable X, and the explanatory variable X refers to the predictors of PD_ij, MMR_ij, RBWL25_ij, PMR_ij, GHE_ij, OOPE_ij, PCHE_ij, NI_ij, NCS_ij, PCGDP_ij, URDI_ij, FNI_ij, GL_ij in a province i for the jth healthcare resource index and UL. Ɛ_ij is a random error term in a province i for the jth index.

**Figure 1:**
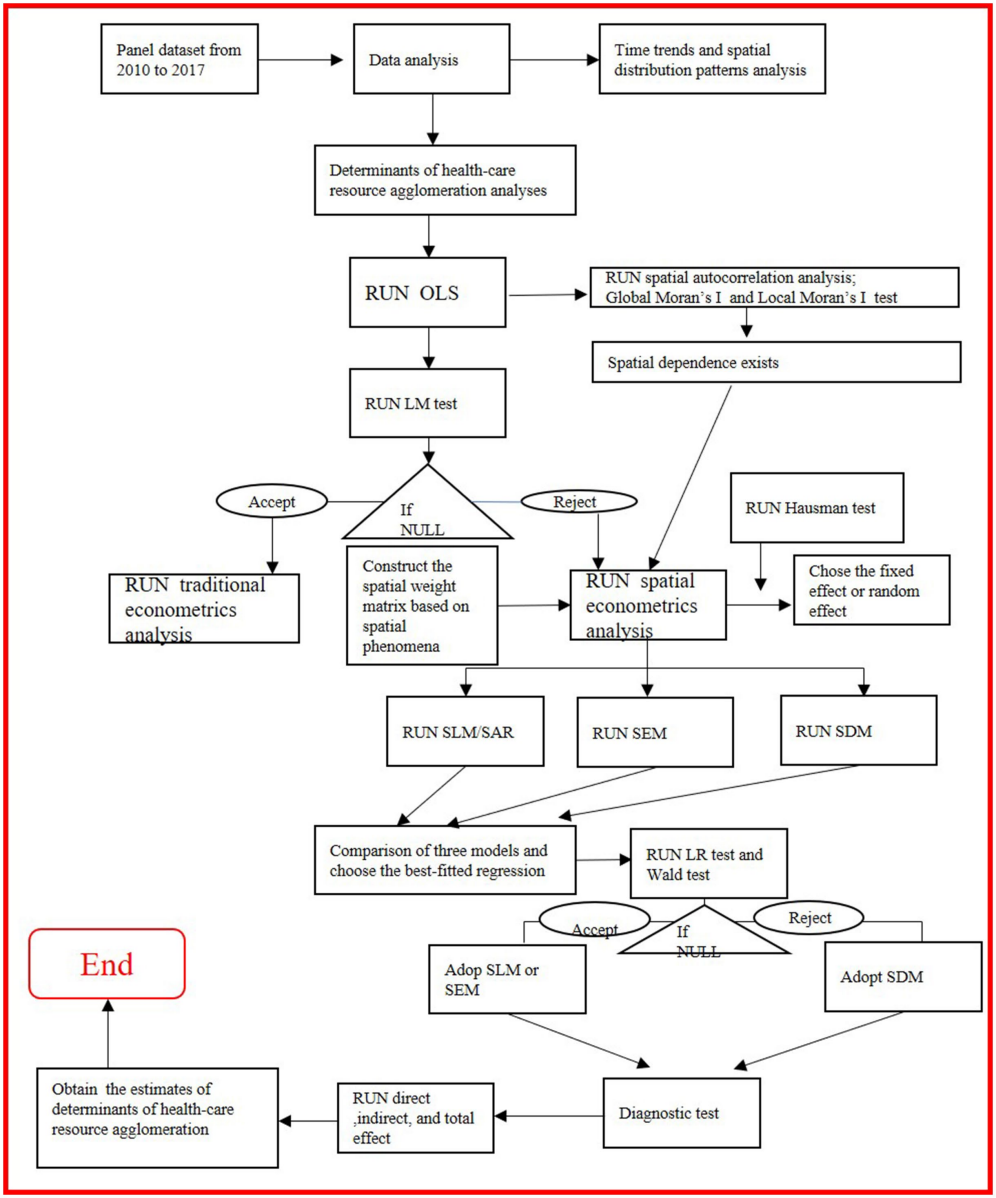
A flowchart of the methodology starting from the source of data-to-data analysis for the study (Source: Author created).

However, upon the confirmation of spatial dependence in the data, the usage of linear regression is not justified, as spatial dependence violates the assumption that observations are independent of each other. Additionally, we tested the multicollinearity in the multivariable linear regression model with variance inflation factor (VIF), and we found that all the values of VIF of some predictors (such as PD_ij, MMR_ij, OOPE_ij, GHE_ij) regressing on the agglomeration indexes of five healthcare resource indicators were more than 5, indicating the existence of the multicollinearity. In the case of spatial dependence in data, spatial regression models such as the spatial lag model, spatial error model, and spatial Durbin model are often used for factors assessment. The spatial regression technique was employed to investigate the relationship between the values of a response variable and the values of explanatory variables. Many scholars have applied spatial techniques in health system research (9, 10), including a study on the COVID-19 pandemic (11). In doing so, for this study, we used longitudinal data to construct a spatial econometric regression model to analyze temporal and spatial evolution trends and to identify the contribution of various factors to healthcare resource agglomeration in China. To make the statistical analysis of the study clearer, a flow chart of the methodology was presented, starting from the source of data-to-data analysis (see Figure 1).

Before we conducted the spatial econometrics analyses, we first constructed the appropriate spatial weight matrix, which illustrates the location information of the geographical units to conduct the spatial econometric analysis of the target geographical units. In general, the construction of a spatial weight matrix can be based on various spatial phenomena, such as proximity, contiguity, similarity, or distance decay, and constructing a spatial weight matrix depending on the nature of the spatial phenomena being analyzed. In this study, we chose a contiguity-based spatial weight matrix, in which observations that share a common boundary or touch each other are assigned a weight of 1, while others are assigned a weight of 0, for our main interest lies in understanding spatial interdependence between adjacent administrative divisions and the contiguity-based spatial weight matrix best represents the spatial relationships between observations in their study area.

Then, we divided spatial econometric models into three types: spatial lag model (SLM) or spatial autoregressive (SAR) model, spatial error model (SEM), and spatial Durbin model (SDM).

#### 2.2.1 SLM (or SAR model)

If the lag term of a dependent variable y is considered to have a spatial autocorrelation, then the dependent variable and its driver model can be expressed as follows:


(3) y=ρWy+Xβ+ε

In Formula (3), where y represents the n × 1 dependent variable. X represents the n × K independent variable. ρ represents the spatial autoregressive coefficient to be estimated. W represents the n × n spatial weight matrix. β represents the K × 1 coefficient of independent variables to be estimated. ɛ represents the n × 1 error term.

#### 2.2.2 SEM

If the spatial dependence of a dependent variable exists in the error disturbance term and is used to measure the effect of the error shock of a dependent variable in neighboring regions on the dependent variable in one region, then the spatial error model can be used in this study. The model is as follows:


(4) y=Xβ+μ

(5) μ=λWμ+v

In Formula (4) and (5), where y represents the n × 1 dependent variable. X represents the n × K independent variable. β represents the k × 1 coefficient of independent variables to be estimated. W represents the n × n spatial weight matrix. Μrepresents a vector of error terms (n × 1) assumed to have autocorrelation. λ represents the spatial lag coefficient to be estimated, which is called the spatial autocorrelation coefficient. ν represents the n × 1 error term.

### 2.3 SDM

The spatial Durbin model (SDM) is a development of the spatial autoregressive model (SAR), in which the effect of spatial lag takes into account the independent and dependent variables. To examine the effect between variables, we chose the SDM as follows:


(6) y=ρWy+Xβ+WXθ+ε

In Formula (6), where y represents the n × 1 dependent variable. X represents the n × k independent variable. ρ represents the spatial autoregressive coefficient to be estimated. W represents the n × n spatial weight matrix. β represents the k × 1 coefficient of independent variables to be estimated. θ represents the k × 1 coefficient of the spatial lag term of independent variables to be estimated. ɛ represents the n × 1 error term.

When θ is zero, the SDM degenerates into the SLM/SAR. When θ + ρβ equals zero, the SDM degenerates into the SEM. **W** is a 31 × 31 spatial weight matrix, and off-diagonal blocks are all zeros. The parameter λ refers to the inter-regional spillover effect caused by the error term of observations. For convenience, we adopted the 0–1 adjacency matrix and set the Hainan and Guangdong provinces as the nearest neighbors.

### 2.4 Statistical criteria for model selection and estimation methods

The selection of the appropriate spatial econometric model was based on two null hypotheses: H0, θ = 0 and H0, θ + ρβ = 0. If both the null hypotheses are rejected, then SDM is selected. We performed the Wald and likelihood ratio (LR) tests under certain conditions to determine the appropriate top-down approach for model selection:

(1) SDM was selected if the findings of the Wald test were significant. (2) SLM/SAR or SEM was selected based on LR statistical values (when θ = 0 or θ + ρβ = 0). (3) SEM was selected if θ + ρβ = 0 based on the LRλ test. (3) SLM/SAR was selected if θ = 0 based on the LRθ test. Otherwise, a non-spatial model (e.g., ordinary least squares regression model) was selected if any probability value was not significant after conducting all the aforementioned tests (see Figure 1, for details).

With regards to estimation methods using spatial regression models, to the best of our knowledge, various methods of estimating spatial panel models have been proposed. Broadly, they fall into two categories: (i) generalized method of moments (GMM) and (ii) quasi-maximum likelihood (QML) estimators. All the models that can be estimated using the Stata command, xsmle, fall into the second category. The exception is the random effects of SEM, whose likelihood function involves a transformation using the Cholesky factors of a rather complicated matrix containing the parameters to be estimated, so that the matrix differentiation is extremely chaotic (12, 13).

### 2.5 Statistical analysis

We used the Geoda1. 14 software^1^ for drawing maps, and Stata SE15. 0 (release 15. StataCorp LLC, College Station, TX, United States) to conduct statistical analyses.

## 3 Results

### 3.1 Time trends and spatial distribution patterns of healthcare resource agglomeration in China from 2009 to 2017

From 2009 to 2017, all the agglomeration indexes of healthcare exhibited a downward trend except for the number of institutions in China (Table 2). Moreover, the average agglomeration indexes for five types of healthcare resources were all >1.0 during the period, indicating the agglomeration of healthcare resources in China remained at a relatively higher level of resource allocation on average during the past 9 years.

**Table 2:** Time trends of healthcare resource agglomeration in China from 2009 to 2017.

Year agglomeration	2009	2010	2011	2012	2013	2014	2015	2016	2017	Annual average agglomeration
Institutions	1.036	1.039	1.06	1.067	1.059	1.035	1.059	1.061	1.059	1.053
Beds	1.028	1.024	1.045	1.001	1.004	1.003	1.002	0.998	0.998	1.011
Doctors	1.111	1.092	1.066	1.038	1.05	1.013	1.014	1.032	1.031	1.050
Technicians	1.088	1.073	1.070	1.007	1.053	1.018	1.017	1.015	1.021	1.040
Nurses	1.091	1.074	1.069	1.017	1.04	1.006	1.032	1.004	1.008	1.038

**Figure 2:**
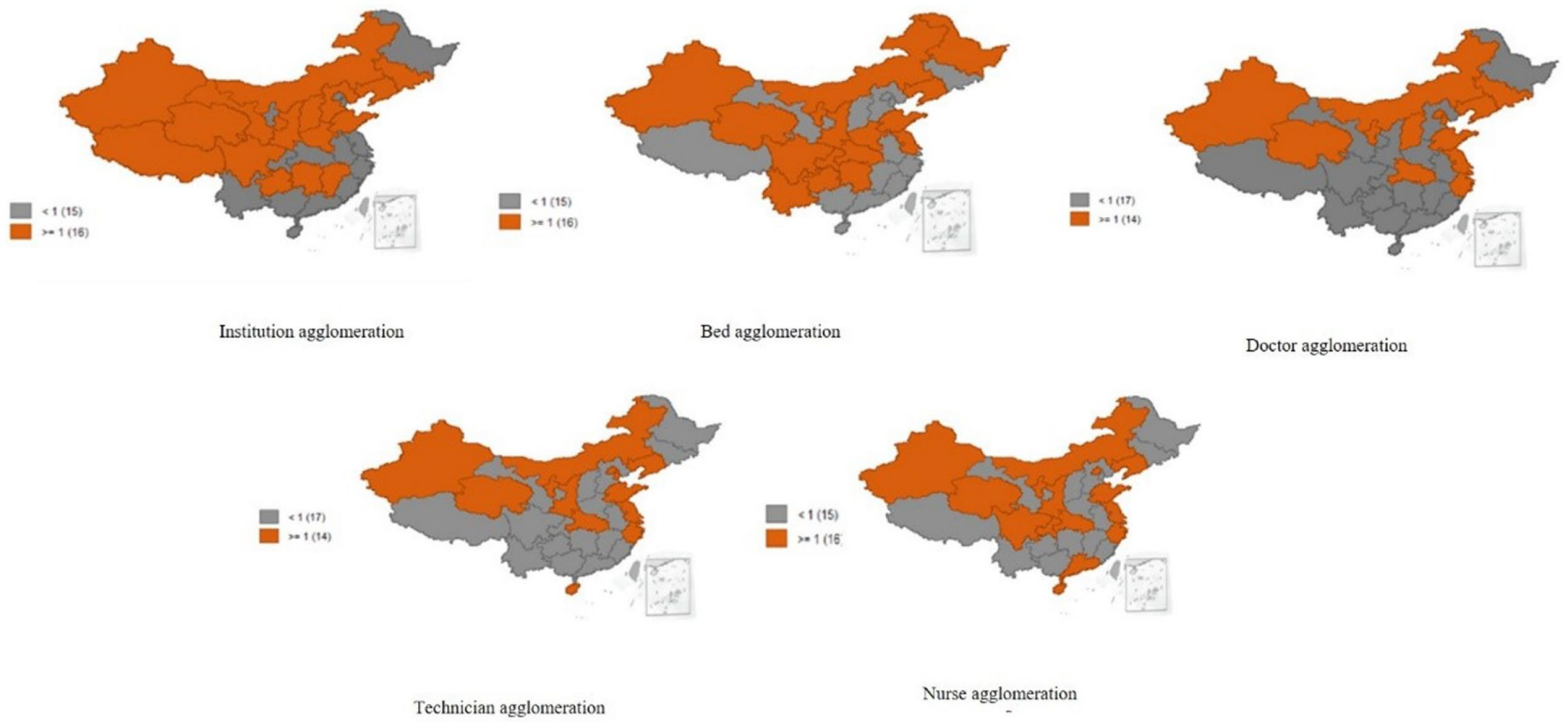
Spatial distribution patterns of healthcare resource agglomeration in China from 2009 to 2017.

In terms of spatial distribution patterns, regional differences in healthcare resource agglomeration were observed from 2009 to 2017. For example, in 2017, we noted a north–south differential spatial distribution of all the agglomeration indexes, with a diagonal angle pattern. Most of the provinces with agglomeration indexes of >1.0 were located in the north of China, accounting for >50% of all provinces. In contrast, other provinces with agglomeration indexes of <1.0 were located in the southwest and southeast of China (Figure 2).

### 3.2 Determinants of healthcare resource agglomeration in China from 2009 to 2017

Before we conducted spatial econometrics analyses, we tested the spatial dependence with Moran’s I index and found the existence of the spatial correlation in the residuals of the OLS estimates (see Table 3). Based on the statistical criteria for selecting spatial econometric models, we chose the SDM for the number of beds, doctors, technicians, and nurses because the Wald test result was significant and the SEM for the number of institutions because the Wald test result was not significant (see Table 4). In addition, based on the Hausman test results, we selected the fixed effect for the number of institutions and the random effect for the number of beds, doctors, technicians, and nurses. Additionally, we used the QML estimation method in this study because we only needed to compute the fixed effect for the number of institutions, which was suitable for SEM analysis, while random effects of the number of beds, doctors, technicians, and nurses were suitable for SDM analyses. All the statistical assumptions required for the abovementioned methods have been satisfied (see Table 4, for details).

**Table 3:** Global spatial correlation test of healthcare resource agglomeration in China from 2009 to 2017.

Year	IA		BA		DA		TA		NA	
	Moran’s I	Z value	Moran’s I	Z value	Moran’s I	Z value	Moran’s I	Z value	Moran’s I	Z value
2009	0.204	2.057^**^	0.163	1.806^**^	0.259	2.878^***^	0.213	2.518^**^	0.159	1.914^**^
2010	0.240	2.419^**^	0.144	1.623	0.269	3.106^***^	0.216	2.584^**^	0.161	2.008^**^
2011	0.174	2.052^**^	0.135	1.625	0.255	2.967^***^	0.206	2.518^**^	0.151	1.924^**^
2012	0.186	2.169^**^	0.033	0.605	0.227	2.544^**^	−0.040	−0.048	−0.009	−0.262
2013	0.198	2.273^**^	0.146	1.551	0.242	2.888^***^	0.176	2.221^**^	0.145	1.862
2014	0.086	1.159	0.172	1.732	0.128	1.605	−0.055	−0.201	−0.100	−0.600
2015	0.198	2.240^**^	0.213	2.072^**^	0.110	1.423	−0.074	−0.391	−0.145	−0.931
2016	0.204	2.275^**^	0.195	1.922^**^	0.175	2.072^**^	−0.080	−0.451	−0.110	−0.670
2017	0.209	2.292^**^	0.263	2.482^**^	0.223	2.595^**^	−0.118	−0.800	−0.134	−0.875

**p < 0.05; ***p < 0.01.

**Table 4:** Spatial econometric regression results for determinants of healthcare resource agglomeration in China from 2009 to 2017.

Variables	SEM		SDM							
	IA		BA		DA		TA		NA	
	Random effect	Fixed effect	Random effect	Fixed effect	Random effect	Fixed effect	Random effect	Fixed effect	Random effect	Fixed effect
PD	0.000052***	−0.000017	0.000009**	0.000015	0.000019**	−0.000040	0.000023***	−0.000042	0.000022**	−0.000042
	(6.664)	(−0.611)	(2.071)	(0.469)	(2.421)	(−0.930)	(3.034)	(−1.032)	(2.421)	(−0.722)
MMR	−0.003743***	−0.001833*	−0.001649**	−0.001627	−0.001002	0.000698	−0.000604	0.001411	−0.001807	−0.000231
	(−5.061)	(−1.774)	(−2.114)	(−1.401)	(−0.923)	(0.444)	(−0.585)	(0.927)	(−1.261)	(−0.109)
BWL25	−0.021498	−0.012576	−0.016970	0.002868	−0.022001	−0.016934	−0.002826	0.004264	−0.018107	−0.023543
	(−1.522)	(−0.922)	(−1.113)	(0.186)	(−1.069)	(−0.817)	(−0.145)	(0.212)	(−0.670)	(−0.838)
PM	−0.004410	−0.002472	0.009227	0.013274*	0.006187	−0.001597	−0.003258	−0.011011	0.002257	−0.000090
	(−0.612)	(−0.339)	(1.351)	(1.692)	(0.618)	(−0.149)	(−0.333)	(−1.022)	(0.176)	(−0.006)
GHE	0.000004	−0.000005	0.000222***	0.000104	0.000062	0.000084	−0.000023	−0.000016	−0.000078	−0.000083
	(0.049)	(−0.062)	(2.615)	(1.236)	(0.537)	(0.725)	(−0.209)	(−0.145)	(−0.500)	(−0.528)
OOPE	0.000008	−0.000011	−0.000113	−0.000201***	0.000058	0.000059	0.000240**	0.000233**	0.000269**	0.000283**
	(0.114)	(−0.174)	(−1.521)	(−2.827)	(0.575)	(0.601)	(2.497)	(2.493)	(1.994)	(2.141)
PCHE	−0.000016	−0.000017*	−0.000045***	−0.000050***	−0.000109***	−0.000120***	−0.000098***	−0.000106***	−0.000129***	−0.000136***
	(−1.635)	(−1.832)	(−4.035)	(−4.648)	(−7.127)	(−8.258)	(−6.740)	(−7.592)	(−6.337)	(−6.921)
NI	0.000002	0.000003	−0.000005	0.000002	0.000001	0.000006	−0.000012	−0.000008	−0.000011	−0.000004
	(0.335)	(0.489)	(−0.660)	(0.279)	(0.135)	(0.616)	(−1.168)	(−0.765)	(−0.743)	(−0.276)
NCS	−0.000000	0.000000	−0.000027***	−0.000026***	−0.000040***	−0.000043***	−0.000042***	−0.000044***	−0.000051***	−0.000051***
	(−0.016)	(0.021)	(−2.919)	(−2.970)	(−3.198)	(−3.655)	(−3.529)	(−3.916)	(−3.029)	(−3.225)
PCGDP	−0.000000	−0.000000	0.000001	0.000001*	0.000000	0.000000	0.000000	0.000000	−0.000000	0.000000
	(−0.603)	(−0.318)	(1.393)	(1.892)	(0.032)	(0.391)	(0.153)	(0.374)	(−0.040)	(0.255)
URDI	0.000003*	0.000003	−0.000003	−0.000003	0.000004	0.000004	0.000005*	0.000005**	0.000006*	0.000007**
	(1.834)	(1.643)	(−1.326)	(−1.604)	(1.503)	(1.577)	(1.790)	(2.085)	(1.786)	(2.221)
FNI	0.000003	0.000001	0.000002	0.000004	0.000007	0.000008*	0.000002	0.000003	0.000000	−0.000002
	(0.879)	(0.521)	(0.654)	(1.210)	(1.402)	(1.677)	(0.428)	(0.600)	(0.009)	(−0.262)
GL	0.092792	0.000000	0.125551***	0.000000	0.122954**	0.000000	0.172930***	0.000000	0.175201**	0.000000
	(1.536)	(.)	(3.772)	(.)	(1.995)	(.)	(3.074)	(.)	(2.518)	(.)
UL	−0.005738**	−0.002265	0.017756***	0.023654***	0.033257***	0.033204***	0.039416***	0.041260***	0.047665***	0.053226***
	(−2.359)	(−0.884)	(8.062)	(8.075)	(10.715)	(8.648)	(12.967)	(10.793)	(11.735)	(9.870)
Wx*PD			−0.000030***	0.000090			−0.000044***	−0.000007	−0.000062***	−0.000168
			(−3.189)	(1.339)			(−2.845)	(−0.080)	(−3.171)	(−1.370)
Wx*MMR			0.006540***	0.004582**	0.010072***	0.011121***	0.011647***	0.010754***	0.013026***	0.015797***
			(4.499)	(2.246)	(5.816)	(6.245)	(6.028)	(3.830)	(4.907)	(4.209)
Wx*BWL25			−0.054356**	−0.050807*						
			(−2.461)	(−1.768)						
Wx*PCGDP							−0.000002**	−0.000003**		
							(−1.998)	(−2.349)		
Hausman test	Chi2 (9)=26.14 Prob>chi2 = 0.026		Chi2 (9)=1.88 Prob>chi2 = 0.993		Chi2 (9)= 2.65 Prob>chi2 < 0.801		Chi2 (9)= 1.55 Prob>chi2 < 0.907		Chi2 (9)=1.19 Prob>chi2 < 0.761	
Wald test	Nesting SAR assumption:Chi2 (13)=31.62 Prob>chi2 = 0.0045 Nesting SEM assumption:Chi2 (13)= 17.95 Prob>chi2 = 0.2797		Nesting SAR assumption:Chi2 (13)=28.48 Prob>chi2 = 0.0123 Nesting SEM assumption:Chi2 (13)=30.94 Prob>chi2 = 0.0057		Nesting SAR assumption:Chi2 (13)=58.72Prob > chi2 < 0.0001 Nesting SEM assumption:Chi2 (13)=27.84 Prob>chi2 = 0.0149		Nesting SAR assumption:Chi2 (13)=48.86Prob > chi2 < 0.0001 Nesting SEM assumption:Chi2 (13)=26.36 Prob>chi2 = 0.0918		SAR assumption:Chi2 (13)=37.34 Prob>chi2 = 0.0007 SEM assumption:Chi2 (13)=26.42 Prob>chi2 = 0.0883	
LR test	Nesting SAR assumption:Chi2 (13)=15.20 Prob>chi2 = 0.0946 Nesting SEM assumption:Chi2 (13)=15.26 Prob>chi2 = 0.2916		Nesting SAR assumption:Chi2 (13)=21.95 Prob>chi2 = 0.0797 Nesting SEM assumption:Chi2 (13)=23.97 Prob>chi2 = 0.0314		Nesting SAR assumption:Chi2 (13)=19.92 Prob>chi2 = 0.0171 Nesting SEM assumption:Chi2 (13)=27.42 Prob>chi2 = 0.0809		Nesting SAR assumption:Chi2 (13)=20.25 Prob>chi2 = 0.0737 Nesting SEM assumption:Chi2 (13)=19.23 Prob>chi2 = 0.0552		SAR assumption:Chi2 (13)=28.262 Prob>chi2 = 0.0011 SEM assumption:Chi2 (13)=17.31 Prob>chi2 = 0.0854	
Rho			0.056039*	0.078552	0.003363*	0.055919	0.000056**	0.014079	0.017276*	0.028693
			(1.870)	(1.085)	(0.853)	(0.864)	(2.001)	(0.224)	(1.869)	(0.439)
ln_phi	2.474874***									
	(8.662)									
Lambda	−0.133749*	−0.141853								
	(−1.685)	(−1.442)								
lgt_theta			−1.219404***		−1.727356***		−1.656658***		−1.472303***	
			(−5.735)		(−10.208)		(−9.937)		(−8.397)	
sigma2_e	0.005605***	0.004847***	0.007017***	0.005924***	0.012690***	0.011106***	0.011523***	0.010097***	0.023030***	0.020168***
	(11.043)	(11.783)	(10.835)	(11.806)	(11.051)	(11.809)	(11.086)	(11.811)	(11.064)	(11.810)
Constant	1.161472***		−0.002249		−0.811778***		−1.073332***		−1.396510***	
	(5.284)		(−0.012)		(−2.999)		(−4.217)		(−4.198)	
Observations	279	279	279	279	279	279	279	279		279
R2	0.664	0.423	0.311	0.044	0.482	0.261	0.510	0.285	0.465	0.247
Units	31	31	31	31	31	31	31	31		31

***p < 0.01; **p < 0.05; *p < 0.10.

For the number of institutions, MMR (*p* < 0.01, t = −5.061) and UL (*p* < 0.05, t = −2.359), and PD (*p* < 0.01, *t* = 6.664) and URDI (*p* < 0.10, *t* = 1.834) were significantly associated with the agglomeration index at 1, 5, 1, and 10% levels, respectively, indicating that MMR and UL had significant negative effects on the agglomeration index for institutions, while PD and URDI had significant positive effects on it. Furthermore, the agglomeration of institutions exhibited a negative interprovincial spatial autocorrelation from 2009 to 2017 (*λ* = −0.134, *p* < 0.10, *t* = −1.685).

For the number of beds, the spatial autoregressive positive coefficient (*ρ* = 0.056) was significant at the 10% level, indicating that the bed agglomeration index exhibited a significant positive spatial correlation between provinces as a whole. PD (*p* < 0.01, *t* = 2.071), MMR (*p* < 0.01, *t* = −2.114), GHE (*p* < 0.01, *t* = 2.615), PCHE (*p* < 0.01, *t* = −4.015), NCS (*p* < 0.01, *t* = −2.919), GL (*p* < 0.01, *t* = 3.772), and UL (*p* < 0.01, *t* = 8.062) exerted a significant effect on bed agglomeration. Moreover, the coefficient of the spatial weight matrix and PD and RBWL25 was less than 0 and significant at the 1% (*p* < 0.01, *t* = −3.189) and 5% level (*p* < 0.05, *t* = −2.461), respectively, indicating that one region PD and RBWL25 had a spillover effect on bed agglomeration in neighboring regions. However, the coefficient of the spatial weight matrix and MMR was more than 0 and significant at the 1% level (*p* < 0.01, *t* = 4.499). Hence, an increase in the PD and BWL25 of one province reduced the bed agglomeration in its adjacent provinces, while an increase in the MMR of one province improved the bed agglomeration in its adjacent provinces.

For the workforce, the spatial autoregressive coefficients for the number of doctors, technicians, and nurses were all positively (all were > 0) significant at 1, 5, and 10% levels, respectively, indicating that the agglomeration of all the three types of the workforce exhibited significant positive spatial correlations among provinces. PD, URDI, GL, and UL exerted a significant positive effect on the agglomeration of the workforce, whereas both PCHE and NCS exerted a significant negative effect on the agglomeration of the three types of the workforce.

The findings concerning the interaction term coefficients of each variable and the spatial weight matrix indicated that MMR had a significantly positive spatial spillover effect on the agglomeration of doctors, medical technicians, and nurses in neighboring provinces. In contrast, PD and PCGDP had a significant negative spatial spillover effect on the agglomeration of the workforce in other provinces.

Table 5 presents the effect decomposition of factors affecting the agglomeration of beds and the workforce based on the SDM. In terms of direct effects, dependent variables of agglomeration of beds and the workforce and their independent variables demonstrated the same connections as shown in the aforementioned SDM in Table 4. In terms of indirect effects, we observed similar results in the aforementioned SDM in Table 4 that MMR exerted a positive spatial spillover effect on the agglomeration of beds and the workforce, whereas PD, RBWL25, and PCGDP exerted a negative spatial spillover effect on the agglomeration of beds and the workforce, i.e., MMR had a positive spatial spillover effect on the agglomeration of beds and the entire workforce, while PD had a negative spatial spillover effect on the agglomeration of beds, technicians, and nurses. RBWL25 had only a negative spatial spillover effect on the agglomeration of beds, and PCGDP had only a negative spatial spillover effect on the agglomeration of nurses. These results were consistent with those of the spatial Durbin regression for determining the agglomeration of healthcare resources in China from 2009 to 2017, as presented in Table 4.

**Table 5:** Decomposition of the spatial spillover effect of beds and workforce agglomeration index in China from 2009 to 2017.

Variables	BA			DA			TA			NA		
	Direct effect	Indirect effect	Total effect	Direct effect	Indirect effect	Total effect	Direct effect	Indirect effect	Total effect	Direct effect	Indirect effect	Total effect
PD	0.000008*	−0.000027***	−0.000018*	0.000020**	<0.000001	0.000020**	0.000023***	−0.000043***	−0.000020	0.000023**	−0.000059***	−0.000037*
	(1.854)	(−2.672)	(−1.691)	(2.394)	(0.194)	(2.345)	(2.978)	(−2.743)	(−1.104)	(2.414)	(−3.062)	(−1.707)
MMR	−0.001629**	0.006460***	0.004831***	−0.001038	0.009876***	0.008838***	−0.000660	0.011459***	0.010799***	−0.001936	0.012704***	0.010767***
	(−2.140)	(4.100)	(2.675)	(−0.980)	(5.670)	(4.223)	(−0.655)	(5.947)	(4.835)	(−1.384)	(4.610)	(3.449)
RBLW25	−0.018831	−0.063129***	−0.081960***	−0.019901	−0.000265	−0.020165	−0.000817	−0.000030	−0.000847	−0.015343	0.000191	−0.015152
	(−1.309)	(−2.653)	(−2.988)	(−1.010)	(−0.151)	(−1.000)	(−0.044)	(−0.028)	(−0.045)	(−0.593)	(0.104)	(−0.592)
PM	0.011546*	0.000602	0.012148*	0.006142	0.000006	0.006148	−0.003337	−0.000017	−0.003354	0.002197	−0.000076	0.002121
	(1.717)	(0.649)	(1.719)	(0.634)	(0.008)	(0.632)	(−0.351)	(−0.032)	(−0.353)	(0.177)	(−0.102)	(0.174)
GHE	0.000225***	0.000013	0.000238***	0.000062	0.000001	0.000063	−0.000023	<0.000001	−0.000023	−0.000078	0.000002	−0.000076
	(2.757)	(0.759)	(2.684)	(0.558)	(0.147)	(0.558)	(−0.216)	(0.079)	(−0.210)	(−0.522)	(0.219)	(−0.514)
OPPE	−0.000108	0.000111	0.000003	0.000064	<0.000001	0.000064	0.000246**	<0.000001	0.000246**	0.000276**	−0.000005	0.000272**
	(−1.453)	(1.264)	(0.025)	(0.631)	(0.049)	(0.625)	(2.561)	(0.004)	(2.566)	(2.057)	(−0.250)	(2.051)
PCHE	−0.000045***	−0.000003	−0.000048***	−0.000109***	> − 0.000001	−0.000111***	−0.000098***	<−0.000001	−0.000099***	−0.000129***	0.000002	−0.000127***
	(−3.922)	(−0.794)	(−3.684)	(−6.942)	(−0.195)	(−6.344)	(−6.536)	(−0.054)	(−5.966)	(−6.169)	(0.242)	(−5.679)
NI	−0.000006	−0.000000	−0.000007	0.000001	<0.000001	0.000001	−0.000013	> − 0.000001	−0.000013	−0.000012	<0.000001	−0.000011
	(−0.779)	(−0.426)	(−0.772)	(0.091)	(0.121)	(0.097)	(−1.229)	(−0.027)	(−1.214)	(−0.797)	(0.185)	(−0.792)
NCS	−0.000027***	−0.000001	−0.000028***	−0.000039***	> − 0.000001	−0.000039***	−0.000041***	> − 0.000001	−0.000041***	−0.000049***	<0.000001	−0.000049***
	(−2.953)	(−0.737)	(−2.901)	(−3.179)	(−0.198)	(−3.106)	(−3.518)	(−0.023)	(−3.465)	(−3.005)	(0.235)	(−2.949)
PCGDP	0.000001	<0.000001	0.000001	<0.000001	<0.000001	<0.000001	<0.000001	−0.000002**	−0.000002	> − 0.000001	<0.000001	> − 0.000001
	(1.431)	(0.636)	(1.411)	(0.059)	(0.042)	(0.061)	(0.181)	(−1.983)	(−1.568)	(−0.012)	(0.047)	(−0.009)
URDI	−0.000003	> − 0.000001	−0.000003	−0.000004	> − 0.000001	−0.000004	−0.000005*	> − 0.000001	−0.000005*	−0.000007*	<0.000001	−0.000006*
	(−1.554)	(−0.629)	(−1.545)	(−1.581)	(−0.149)	(−1.579)	(−1.876)	(−0.028)	(−1.864)	(−1.867)	(0.240)	(−1.860)
FNI	0.000002	<0.000001	0.000002	0.000007	<0.000001	0.000007	0.000002	<0.000001	0.000002	<0.000001	> − 0.000001	<0.000001
	(0.562)	(0.353)	(0.563)	(1.396)	(0.143)	(1.391)	(0.452)	(0.037)	(0.454)	(0.045)	(−0.013)	(0.045)
GL	0.110733***	0.006293	0.117026***	0.119714**	0.001798	0.121512**	0.170212***	0.000219	0.170432***	0.171661***	−0.002502	0.169159**
	(3.516)	(0.807)	(3.440)	(2.028)	(0.227)	(1.995)	(3.130)	(0.023)	(3.117)	(2.576)	(−0.230)	(2.550)
UL	0.017000***	0.000955	0.017955***	0.033172***	0.000407	0.033579***	0.039370***	0.000063	0.039433***	0.047562***	−0.000731	0.046831***
	(7.960)	(0.822)	(7.246)	(10.761)	(0.198)	(8.884)	(13.085)	(0.029)	(11.363)	(11.814)	(−0.255)	(9.856)

***p < 0.01; **p < 0.05; *p < 0.10.

## 4 Discussion

The findings of this study revealed a downward trend in the healthcare resource agglomeration index, except for the number of institutions that remained at a relatively higher level of resource allocation on average in China from 2009 to 2017. This finding can be attributed to China’s health reform efforts since 2009 to optimize the allocation of healthcare resources in China. Each province increased its healthcare resources through various means, such as by expanding its healthcare expenditures, building hospitals, increasing the number of beds, implementing public hospital reforms, and improving primary health services. However, the increasing number of healthcare resources remained inadequate for the growing population, resulting in declining agglomeration indexes for the number of beds, doctors, technicians, and nurses. Moreover, the healthcare resource agglomeration index exhibited a north–south differential distribution pattern across provinces. In particular, the distribution of the healthcare resource agglomeration index in the northwest, north, and northeast regions was superior to that in the southwest and southeast regions. This may be due to China’s preferential healthcare resource input into western regions in light of the implementation of China’s western development strategies since the 2000s. In addition, this finding might be attributable to the lower population in western regions than in eastern and southern regions. These factors led to a high agglomeration index in western China. This finding is in line with those of some previous studies (14–16).

PD, GHE, URDI, GL, and UL all had positive significant effects on the agglomeration of beds. However, PD, BWL25, and PCGDP had negative spatial spillover effects, and MMR had a positive spatial spillover effect on both beds and the workforce in neighboring regions.

Regarding PD, for a province, having a larger population density means inadequate access to healthcare to meet the needs of residents there for health services, which stimulates an increase of government investment in healthcare resources and an improvement of the agglomeration of healthcare resources in that province. Similarly, as part of the total health expenditure, GHE ensures that the government provides adequate healthcare resources to individuals. The supply of healthcare resources effectively increased with an increase in GHE. A previous study reported that the average annual growth rate of *per capita* GHE increased by 10.68%, increasing from **¥**191.08 in 2009 to **¥**526.95 in 2018, which further increased the probability of the concentration and agglomeration of healthcare resources across various provinces in China (17). However, factors such as differences in GHE and URDI imbalances, economic development, and the segmentation of healthcare resources across various regions have led to large differences in healthcare resource agglomeration in China (18–20).

In terms of GL, in the central and western regions, the higher the healthcare resource agglomeration index was, the more reasonable the healthcare resource allocation in those areas. GL was demonstrated to play a crucial role in healthcare resource agglomeration (21, 22). This finding can be explained by China’s focus on the coordinated development of regional economies to narrow economic and social gaps among the eastern, central, and western regions in light of the 12th Five-Year Plan and 13th Five-Year Plan since the implementation of the new medical reforms. The government had gradually increased healthcare investment in the western and central regions through various policy-related measures, including redirecting investment, capital arrangements, subsidy provisions, and supplying large-scale medical equipment (23), leading to the accumulation of beds and equipment in these regions. Moreover, the government has enhanced the agglomeration of the workforce in the western and central regions through various efforts, including the implementation of the east–west pairing health assistance programs (24, 25) and the application for special fiscal funds to the Ministry of Health, the establishment of the western health talent training project, the provision of aid to projects in Xinjiang and Tibet, and the development of policies to emphasize health talent development. The two-way mechanism of balancing the healthcare workforce between the eastern and western provinces improved the agglomeration of resources in corresponding neighboring regions (26).

In this study, UL was observed to exert a positive effect on the agglomeration of beds and healthcare human resources. This is because UL can boost economic growth by expanding demand and prompt the government to devote more energy to healthcare services and provide more beds, doctors, technicians, and nurses, thus promoting an increase in their agglomeration.

Notably, PD exerts negative spillover effects on the agglomeration of the beds and health workforce in terms of the interregional two-way flow of healthcare resources. As mentioned above, PD causes the health input of government and agglomeration of healthcare resources in one province and inevitably attracts the residents interprovincial-seeking more access to healthcare services and affects remaining agglomeration of healthcare resources in other adjacent provinces comparatively. Additionally, for one province, the higher the PCGDP is, the more it spends on healthcare, such as expanding the number of health institutions’ beds and increasing salaries of the health workforce. When the medical institutions, salary, and career development opportunities in one region are attractive, the inflow of the healthcare workforce from neighboring regions accelerates, thus reducing the agglomeration of the health workforce in the neighborhood. This finding is in accordance with those of some previous studies reporting that the spillover effect of PCGDP weakened the agglomeration of the workforce in adjacent regions (27–29).

In this study, PCHE, NCS, and MMR exerted significant negative effects on the agglomeration of institutions, beds, and the workforce. MMR also exerted a positive spatial spillover effect on the agglomeration of beds and the workforce in neighboring provinces. The implementation of the new medical reform in 2009, the promotion of urbanization, and the aging of the population have caused a gradual increase in China’s PCHE to meet the growing needs for healthcare services in the country. As a crucial component of total health expenditures consisting of GHE, SHE, and PCHE, the increase in PCHE in one region increases GHE, resulting in a decline in the agglomeration of healthcare resources in that region. Moreover, the increases in PCHE aggravate the burden of residents and decrease residents’ consumption in other aspects, thus restricting the health service needs of residents and the economic development of the country and reducing the agglomeration of beds and the workforce. To prevent this situation, the Chinese government has focused on alleviating the medical care burden of residents by implementing various medical reform policies, such as the New Rural Cooperative Medical Insurance Scheme and the Urban Residents Basic Medical Insurance. Thus, each province has made considerable efforts to reduce PCHE through various measures such as guaranteeing quality and cutting down on unnecessary expenses, reducing drug prices, developing medical payment reforms to control the growth of unreasonable PCHE, meeting the health service needs of residents, and enhancing government investment in healthcare resources to develop the economy in the region (30, 31).

For NCS, the number of college students reflects the extent to which one province invests in higher education, thus under a certain limit of government spending, more spending on education indicates that more investment in health is virtually squeezed out, resulting in a decline in agglomeration of beds and health-human resources. This finding is also consistent with prior literature (32, 33). Through the same crowd-out mechanism of PCHE mentioned above, the MMR, widely accepted as a key indicator of health and socioeconomic development, also aggravates the burden of residents and decreases residents’ consumption and government spending in other aspects, thus reducing the agglomeration of institutions and beds. Interestingly, MMR had a positive spillover effect on the agglomeration of beds and the health workforce in the neighborhood. This may be due to the “lesson-learning” effect, meaning that the reduction of MMR in one province compels neighboring provinces to implement stronger measures to reduce MMR in their regions, which results in their meeting the unmet health needs of residents and increasing the government health investment and agglomeration of healthcare resources. Some previous studies had supported one region’s MMR positive spatial spillover effect on the agglomeration of healthcare resources in the adjacent regions (34, 35).

This study has some limitations that should be addressed. First, the longitudinal data used in this study can only reflect the healthcare resource allocation status at the cut-off point of this work and does not reflect the entire picture. Therefore, a future study on changes in healthcare resource allocation, especially changes due to COVID-19 from 2020, along with comparisons with the present study, can be carried out when the data from 2017 to the present are available. Second, the calculation of the agglomeration index depends on the population in one region instead of its geography. Different results might be obtained when using the geography-based agglomeration index; this should be examined in future studies. Third, we conducted spatial economic analysis by using an adjacency spatial matrix method. Because the construction of the spatial weight matrix includes adjacency, inverse distance, economic characteristics, and nested matrices (36, 37), using different weight matrices can produce different spatial economic regression results. Therefore, a follow-up study should perform a spatial econometric analysis using two other spatial weight matrices and compare their results with those of the present study.

To balance regional differences in the agglomeration index of healthcare resources between the west and east of China, some region-specific measures should be implemented. In light of the national-level China Central Rise and China Western Development strategies, the government has been increasing the number of beds and the healthcare workforce in the central and western regions to achieve the goal of equalizing the east–west allocation of health resources. The government should focus on optimizing the internal distribution structure of healthcare resources in western regions. In accordance with the government’s latest health planning, some efforts, such as strictly controlling the number and scale of public hospitals and implementing a quality-oriented institutional development model that improves quality and efficiency instead of the rough quantity-oriented model, are required to reduce the agglomeration of institutions. Moreover, the allocation of health resources in some eastern regions should be optimized. Because healthcare resources in some eastern provinces, especially the workforce, were not adequately agglomerated, the agglomeration index in these regions was <1.0. Therefore, to increase the average level of healthcare resource agglomeration to meet the increasing needs of those regions, the government should increase the number of beds and the workforce in eastern provinces, especially in some key developed megacities with limited healthcare resources or non-central cities in the metropolitan area (38).

Considering the positive effects of PD, GHE, GL, and UL on the agglomeration of institutions, beds, and health workforce, the government should focus on consistently increasing PD, GHE, URDI, and UL. Moreover, the government should consider the negative spillover effects of PD and PCGDP on the workforce of neighboring regions and limit their development to prevent the imbalanced agglomeration of the health workforce. Correspondingly, a cross-regional consortium should be established to rationally allocate the healthcare workforce: provinces with a relatively low workforce, PD or PCGDP could coordinate with those with abundant resources and higher PD or PCGDP for the introduction and training of the healthcare workforce to maintain their balanced agglomeration.

To increase the agglomeration of healthcare resources, the government should consider the negative effect of MMR in one province and a spatial spillover effect in its adjacent provinces. For example, the government can place a high priority on maternal and child health (MCH) services and integrate vertical programs (e.g., family planning) related to MCH and alleviate the medical burden of individual residents (39). In addition, considering the positive spillover effect of MMR on the agglomeration of the beds and health workforce, the government should also construct and strengthen interprovincial cooperation in health planning and jointly formulate health development strategies to reduce MMR. Each province and its adjacent provinces should achieve a synchronous goal in terms of the agglomeration of the healthcare workforce, reduce regional differences in MMR and healthcare resource allocation, and guarantee the common sustainable development of people’s health in two regions.

The data in our study were derived from secondary statistical yearbooks, which were officially published by the Chinese government. These yearbooks provide specific indicators for the 31 provinces (municipalities or autonomous regions) in China. However, it is important to note that some key variables, such as the cost of medical services, mortality rates, the density of older adult populations, the prevalence of certain diseases in each region, and the overall level of education, were only available for a subset of provinces (municipalities or autonomous regions) and not uniformly across all provinces in China for the period spanning from 2009 to 2017. To address this issue, we plan to incorporate these omitted indicators in our future research projects. This will enhance the comprehensiveness of our study and provide a more in-depth understanding of the healthcare agglomeration time trends and the determinant factors within China’s diverse provinces (municipalities or autonomous regions).

## 5 Conclusion

The agglomeration of healthcare resources was observed to remain at a relatively higher level of resource allocation on average in China from 2009 to 2017. However, some north–south differential distributions in this agglomeration were noted across provinces. Using spatial econometric models, we identified that PD, GHE, URDI, GL, and UL all had positive significant effects on the agglomeration of beds, whereas both PCHE, NCS, and MMR expense had significant negative effects on the agglomeration of institutions, beds, and the workforce. In addition, PD and PCGDP in one province had negative spatial spillover effects on the agglomeration of beds and the workforce in neighboring provinces. However, MMR had a positive spatial spillover effect on the agglomeration of the beds and workforce in those regions. The findings of this study can help the Chinese government and other developing countries develop appropriate measures to balance regional disparities in the agglomeration of healthcare resources across administrative regions.

## Data availability statement

The raw data supporting the conclusions of this article will be made available by the authors, without undue reservation.

## Author contributions

ED: Conceptualization, Funding acquisition, Supervision, Writing – original draft, Writing – review & editing. XS: Formal analysis, Investigation, Methodology, Software, Writing – review & editing. YX: Data curation, Formal analysis, Investigation, Methodology, Writing – original draft. YW: Investigation, Methodology, Writing – original draft. TW: Conceptualization, Data curation, Funding acquisition, Project administration, Resources, Supervision, Writing – review & editing. WG: Conceptualization, Funding acquisition, Project administration, Resources, Supervision, Writing – review & editing.

## Footnotes

^1^http://geodacenter.Github.io/

## References

[1] Sha L Jian G Yongliang Y Yaru W. The study of health resources allocation efficiency in Shaanxi province based on DEA method. Chin Health Serv Manag. (2012) 29:572–4.

[2] Gu J. A spatial study on the allocation of healthcare resource. Chin J Health Stat. (2014) 31:21–3.

[3] Horev T Pesis-Katz I Mukamel DB. Trends in geographic disparities in allocation of health care resources in the US. Health Policy. (2004) 68:223–32. doi: 10.1016/j.healthpol.2003.09.011, PMID: 15063021

[4] Qiong T Ping W. An analysis of the gap of urban-rural basic medical and health resources allocation based on GE index method–taking Chengdu as an example. Consum Econ. (2013) 29:28–31.

[5] Xie X Wu Q Hao Y Yin H Fu W Ning N. Identifying determinants of socioeconomic inequality in health service utilization among patients with chronic non-communicable diseases in China. PLoS One. (2014) 9:1–14. doi: 10.1371/journal.pone.0100231, PMID: 24960168 PMC4069022

[6] Li C Zeng L Dibley MJ Wang D Pei L Yan H. Evaluation of socio-economic inequalities in the use of maternal health services in rural western China. Public Health. (2015) 129:1251–7. doi: 10.1016/j.puhe.2015.07.002, PMID: 26256911

[7] Pan L Wang C Cao X Zhu H Luo L. Unmet healthcare needs and their determining factors among unwell migrants: a comparative study in Shanghai. Int J Environ Res Public Health. (2022) 19:5499. doi: 10.3390/ijerph19095499, PMID: 35564894 PMC9103782

[8] Guo Q Luo K Hu R. The spatial correlations of health resource agglomeration capacities and their influencing factors: evidence from China. Int J Environ Res Public Health. (2020) 17:8705. doi: 10.3390/ijerph17228705, PMID: 33238597 PMC7700579

[9] Tiwary B Nilima N Kumar A Kaushik S Khan MA Pandey PK. Spatial evaluation of pregnancy loss among child-bearing women in India. GeoJournal. (2022) 87:3815–26. doi: 10.1007/s10708-021-10464-9

[10] Singh M Alam MS Majumdar P Tiwary B Narzari H Mahendradhata Y. Understanding the spatial predictors of malnutrition among 0–2 years children in India using path analysis. Front Public Health. (2021) 9:667502. doi: 10.3389/fpubh.2021.667502, PMID: 34395360 PMC8362662

[11] Nilima N Kaushik S Tiwary B Pandey PK. Psycho-social factors associated with the nationwide lockdown in India during COVID-19 pandemic. Clin Epidemiol Glob Health. (2021) 9:47–52. doi: 10.1016/j.cegh.2020.06.010, PMID: 32838060 PMC7324916

[12] Belotti F Hughes G Mortari AP. Spatial panel-data models using Stata. Stata J. (2017) 17:139–80. doi: 10.1177/1536867X1701700109

[13] Anselin L Anselin L. The maximum likelihood approach to spatial process models. Spatial Econ. (1988) 1:57–80. doi: 10.1007/978-94-015-7799-1_6

[14] Dong EH Li GH Cai YY Wang TA. Review on regional difference in healthcare resource allocation. Chin Health Resour. (2016) 5:390–3.

[15] Li JG Li ZY. The regional differences of public health service and the countermeasures. Rev Econ Res. (2012) 34:62–7.

[16] Zhang N Sun XJ Li C Wang X Liu K. Analyzing the equity of health resources allocation in China based on Theil index. Chin Health Serv Manage. (2014) 2:88–91.

[17] Feng CY Liang RH Jiang XM. Analysis of the government health expenditure in the first decade of Chinese new medical reform(2009-2018):Xinjiang Uygur autonomous region as an example. Risk Manag Healthc Policy. (2020) 13:387–95. doi: 10.2147/RMHP.S252652, PMID: 32523386 PMC7234958

[18] Li D Zhou Z Si Y Xu Y Shen C Wang Y. Unequal distribution of health human resource in mainland China:what are the determinants from a comprehensive perspective? Int J Equity Health. (2018) 17:1–2. doi: 10.1186/s12939-018-0742-z 29486791 PMC5830142

[19] Zhou Q Ni J. Perspective on the equity of health human resources allocation in Sichuan Province. Soft Sci Health. (2017) 2:18–22.

[20] Song S Yuan B Zhang L Cheng G Zhu W Hou Z. Increased inequalities in health resource and access to health care in rural China. Int J Environ Res Public Health. (2019) 16:49. doi: 10.3390/ijerph16010049 PMC6338945 30585243

[21] Baicker K Chandra A. Understanding agglomerations in health care In: Baicker K, editor. Agglomeration economics. Chicago: University of Chicago Press (2010). 211–36.

[22] Wan S Chen Y Xiao Y Zhao Q Li M Wu S. Spatial analysis and evaluation of medical resource allocation in China based on geographic big data. BMC Health Serv Res. (2021) 21:1–8. doi: 10.1186/s12913-021-07119-3 34641850 PMC8508408

[23] Lou K Liu B Wang Y. Surveying the status of medical equipment in county and Township's medical institutions in Western. China Chin Health Serv Manage. (2009) 26:617–8.

[24] Lu ZH Meng QY Wang Y. Equity in basic health human resource allocation in China before and after health reform. Chin J Public Health. (2017) 33:1086–868.

[25] Wang CL Liu XP Xie ZY. Development strategy for prefecture-level public hospital in mid-west area under the new round health refrom in China. Chin J Health Policy. (2009) 2:32–6.

[26] Wang XF Hu B. Research on the temporal trend of Chinas driving factors of economy and the differentiation of regional factors–based on the perspective of the supply-side structural factor restructuring. Economist. (2016) 11:26–36.

[27] Zhao AF Wen-Lin FU School B. Regional factor income mobility in China and its influencing elements in the high-quality economic development. East China Econ Manage. (2019) 33:47–53.

[28] Zhou JL Gang LI. Distance of regional economic development: new economic geography, element flow and economic policy. Econ Theory Bus Manag. (2008) 16:29–34.

[29] Wu X Zhang Y Guo X. Research on the equity and influencing factors of medical and health resources allocation in the context of COVID-19:a case of Taiyuan, China. Healthcare. (2022) 10:1319. doi: 10.3390/healthcare10071319 35885847 PMC9324996

[30] Fang M Shao-Long WU. Did "new medical and healthcare systems reform" bring medical expenses down:based on the trace data of CHARLS of Gansu & Zhejiang. J Beijing Adm Inst. (2017) 6:18–27.

[31] Dai SY Ai-Jun XU. Source analysis on total health expenditure in Jiangsu Province from 2010 to 2017. Soft Sci Health. (2019) 33:57–61.

[32] Barnett MS Brooks MR. China: Does government health and education spending boost consumption? Washington, DC: International Monetary Fund (2010).

[33] Psacharopoulos G Patrinos HA. Returns to investment in education: a decennial review of the global literature. Educ Econ. (2018) 26:445–58. doi: 10.1080/09645292.2018.1484426

[34] Owusu PA Sarkodie SA Pedersen PA. Relationship between mortality and health care expenditure: sustainable assessment of health care system. PLoS One. (2021) 16:e0247413. doi: 10.1371/journal.pone.0247413, PMID: 33626059 PMC7904168

[35] Odekunle FF. Maternal mortality burden: the influence of socio-cultural factors. Int J Health Sci Res. (2016) 6:316–24.

[36] Dong K Guo L. Research on the spatial correlation and spatial lag of COVID-19 infection based on spatial analysis. Sustain For. (2021) 13:12013. doi: 10.3390/su132112013

[37] Safi A Wang QS Wahab S. Revisiting the nexus between fiscal decentralization and environment: evidence from fiscally decentralized economies. Environ Sci Pollut Res. (2022) 29:58053–64. doi: 10.1007/s11356-022-19860-1 35364787

[38] Sheng L. Research on status and countermeasures of medical and health resources allocation in China. Dev Res. (2021) 38:68–76.

[39] Acharya K Subedi D Acharya P. Health facility readiness to provide integrated family planning, maternal and child health(FPMCH)services in Nepal: evidence from the comprehensive health facility survey. PLoS One. (2022) 17:e0264417. doi: 10.1371/journal.pone.0264417, PMID: 35213614 PMC8880709

